# Psychometric validation of the Romanian Scenario-Based Dieting Self-Efficacy Scale in adults with overweight and obesity

**DOI:** 10.3389/fnut.2026.1911825

**Published:** 2026-09-01

**Authors:** Diana Draia, Salomeia Putnoky, Ioana Cristina Olariu, Costela Lacrimioara Serban

**Affiliations:** 1 Doctoral School, Center for Translational Research and Systems Medicine, “Victor Babes” University of Medicine and Pharmacy, Timisoara, Romania; 2 Department of Microbiology, Discipline of Hygiene, “Victor Babes” University of Medicine and Pharmacy, Timisoara, Romania; 3 Department of Pediatrics, "Victor Babes" University of Medicine and Pharmacy, Timisoara, Romania; 4 Department of Functional Sciences, Discipline of Public Health, Center for Translational Research and Systems Medicine, “Victor Babes” University of Medicine and Pharmacy, Timisoara, Romania

**Keywords:** body image, construct validity, dieting self-efficacy, eating behaviour, excess weight, factorial structure, obesity, overweight

## Abstract

**Introduction:**

Overweight and obesity require sustained changes in eating behaviour, yet long-term adherence to dietary recommendations remains difficult. Dieting self-efficacy, defined as confidence in managing eating-related challenges, is an important self-regulatory construct in weight management. This study aimed to translate and evaluate the psychometric properties of the Romanian version of the Scenario-Based Dieting Self-Efficacy Scale (DIET-SE) in adults with overweight and obesity.

**Methods:**

A total of 509 adults from the general Romanian population completed an online survey between February and April 2026. For the validation analyses, 243 participants with BMI ≥ 25 kg/m^2^ were included. The sample was randomly divided into two independent subsamples for exploratory factor analysis (EFA; *n* = 123) and confirmatory factor analysis (CFA; *n* = 120). Internal consistency was assessed using Cronbach’s alpha, test–retest reliability using the intraclass correlation coefficient (ICC), and construct validity through associations with intuitive eating, BMI, perceived stress, body image, self-rated health, and fruit and vegetable intake.

**Results:**

EFA supported a three-factor structure consistent with the original instrument, comprising Social and Internal Factors, Negative Emotional Events, and High-Caloric Food, which explained 52.9% of the total variance. CFA provided acceptable support for this structure, with CFI = 0.935, TLI = 0.908, SRMR = 0.058, and RMSEA = 0.090. Internal consistency was good for the total scale (Cronbach’s alpha = 0.872) and acceptable to good for the subscales. Test–retest reliability was excellent (ICC = 0.942). The DIET-SE total score showed positive correlations with the Intuitive Eating Scale-2 total score and with the Reliance on Hunger and Satiety Cues and Body–Food Choice Congruence subscales. Lower dieting self-efficacy was associated with higher BMI, greater perceived stress, larger perceived body shape, and greater discrepancy between perceived and desired body shape, while higher scores were associated with better self-rated health and higher fruit and vegetable intake.

**Conclusion:**

The Romanian version of the DIET-SE demonstrated satisfactory validity and reliability in adults with overweight and obesity and may be useful for assessing eating-related self-efficacy in research and clinical contexts.

## 1 Introduction

Overweight and obesity are major public health concerns, and their management relies substantially on sustained changes in eating behaviour and other lifestyle-related behaviours (1, 2). However, long-term adherence to dietary recommendations remains difficult, which highlights the need to understand the psychological mechanisms that support behaviour change in adults with excess weight (1).

Self-efficacy is a central construct in social cognitive theory and refers to an individual’s perceived ability to perform a specific behaviour despite barriers (3). In weight management, dieting self-efficacy reflects confidence in regulating eating behaviour in challenging situations, such as exposure to high-caloric foods, social pressure, hunger, fatigue, or negative emotional states. Previous evidence indicates that self-efficacy is an important self-regulatory mechanism in obesity interventions and that dietary self-efficacy can be improved through targeted behaviour-change strategies (1, 4).

The Scenario-Based Dieting Self-Efficacy Scale (DIET-SE) was developed as a brief instrument for assessing confidence in managing eating-related temptation situations (5). The scale includes 11 items covering three dimensions: high-caloric food temptations, social and internal factors, and negative emotional events. Following its original development, the DIET-SE has been translated and psychometrically evaluated in Polish, Mexican Spanish, and Turkish populations. These studies generally supported the original three-dimensional structure and reported acceptable internal consistency, suggesting that the instrument may retain its conceptual organization across different cultural settings (5–8).

Since self-efficacy is behaviour-specific and context-sensitive, cross-cultural adaptation and psychometric validation are necessary before using the instrument in Romanian populations. Therefore, this study aimed to translate and evaluate the psychometric properties of the Romanian version of the DIET-SE in adults with overweight and obesity.

## 2 Methods

The Scenario-Based Dieting Self-Efficacy Scale was developed by Stich et al. (5) and includes 11 items. This scale fulfilled the need for an instrument suitable for both research and clinical practice, with good respondent acceptability, clear wording for patients, and straightforward interpretation by investigators based on the scoring system proposed by Stich (5). The dimensions captured by the instrument, resistance to high-caloric food temptations, coping with social and internal factors, and coping with negative emotional events are easy to understand and may support timely and appropriate interventions.

Written permission to translate, culturally adapt, validate, and publish the Romanian version of the DIET-SE was obtained from Professor Bärbel Knäuper, one of the developers of the original instrument.

The DIET-SE was translated and culturally adapted following a forward–backward translation procedure. First, two independent translators produced Romanian versions of the original English scale. The two translations were compared and reconciled into a preliminary Romanian version. This version was then back-translated into English by an independent translator. The back-translated version was compared with the original instrument to identify discrepancies in meaning, and minor wording adjustments were made where necessary. The preliminary Romanian version was pretested in a group of 10 volunteers, who assessed item clarity, comprehensibility, and semantic equivalence. Based on their feedback, minor revisions were introduced, resulting in the final Romanian version (Supplementary Material) used for data collection.

A total of 509 individuals from the general population participated in the study, between February and April 2026, as part of a larger project conducted at the Victor Babeș University of Medicine and Pharmacy in Timișoara, Romania. Participants were recruited through direct invitations by members of the research team, both face-to-face and online, using professional and personal networks. Participants were also encouraged to distribute the survey link within their wider social circles, resulting in a non-probability snowball sampling approach. The online questionnaire required completion of all relevant items before submission; therefore, the analytical dataset contained no missing observations, and no imputation was performed.

In addition to the DIET-SE items, several other variables were collected and included in the analyses, namely gender, age, self-reported weight and height, self-rated health status, and perceived stress level. Self-rated health status was assessed using five ordinal categories, ranging from excellent to poor. Perceived stress was rated on a 10-point scale, with higher scores indicating higher perceived stress. Perceived and desired body image were assessed using the Pictorial Body Image Instrument developed by Collins (9), which consists of nine images depicting body shapes from very thin to severely obese. Participants also reported their estimated average fruit and vegetable intake during the previous 3 days. This variable was recorded on an ordinal scale with six response categories (1 serving or less, 2 servings, 3 servings, 4 servings, 5 servings, more than 5 servings per day), corresponding to increasing numbers of servings. Higher scores indicated higher fruit and vegetable intake. Educational attainment was recorded using ISCED categories in 3 levels, equivalent to ISCED 3 or 4, ISCED 5 or 6, ISCED 7 or 8. Household net income level was recorded as 6 incremental levels. Participants could also select “prefer not to answer.” Participants also completed the Intuitive Eating Scale-2 (IES-2), which was previously validated in Romanian by Vintilă et al. (10).

**Figure 1:**
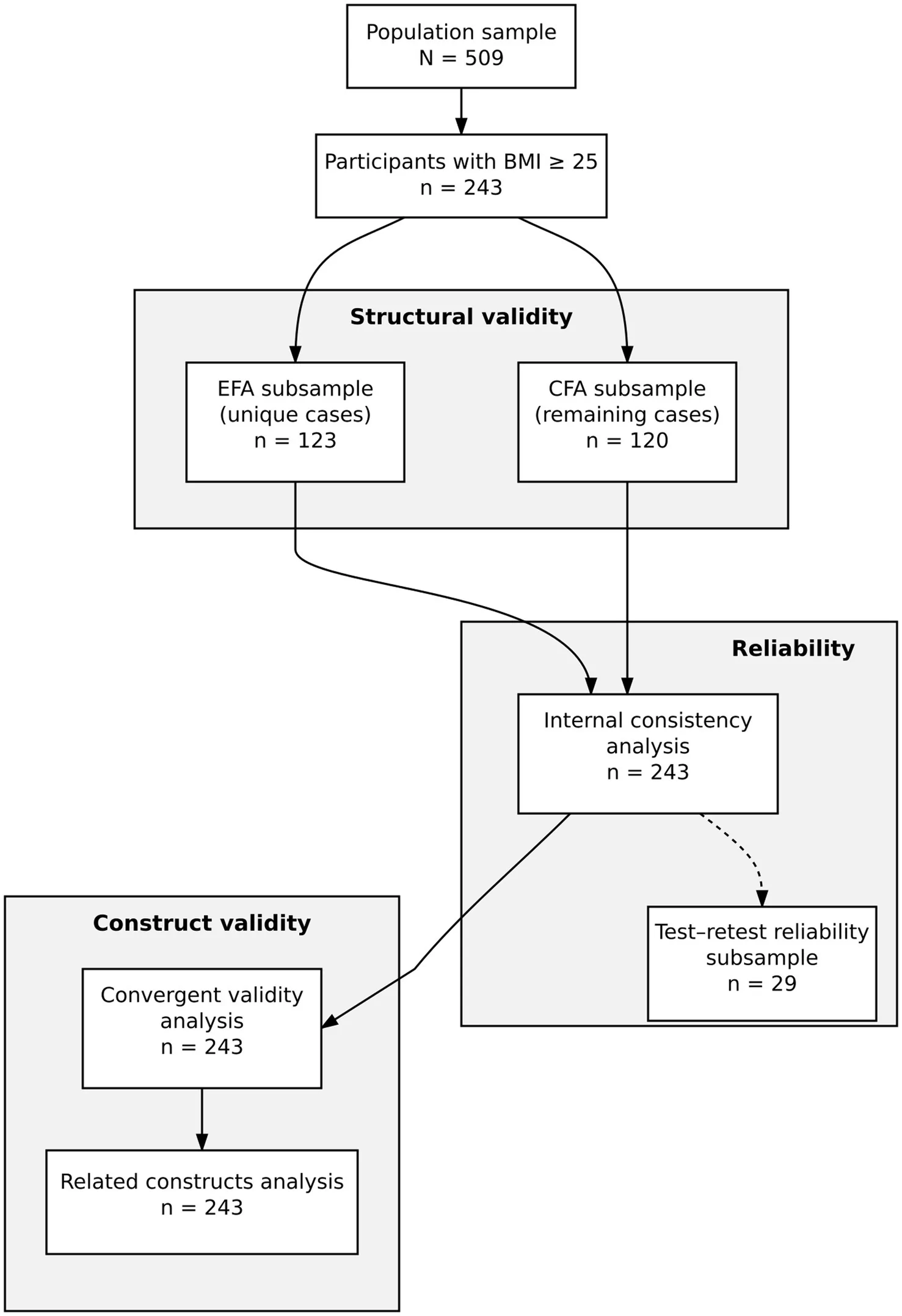
Flowchart of participant selection, sample allocation, and validation analyses.

For the purpose of this validation study, only participants with BMI ≥ 25 kg/m^2^ (*n* = 243) were included. Height and weight were self-reported and were used to calculate BMI (Figure 1).

To examine potential common method bias, Harman’s single-factor test was conducted using the complete validation sample. All item-level self-report measures were entered simultaneously into an unrotated principal component analysis constrained to a single factor. The proportion of total variance explained by the single factor was examined, with a value below 50% considered as indicating that no single factor accounted for the majority of the variance.

Structural validity was assessed using both exploratory factor analysis (EFA) and confirmatory factor analysis (CFA). For this purpose, the sample was divided into two fully independent subsamples: 123 individuals were included in the EFA sample and 120 individuals in the CFA sample. We expected a factor structure similar to the one described by Stich (5). The sample size was considered adequate for factor analysis, as it ensured above 10 participants per item, which is commonly regarded as acceptable in questionnaire validation studies (Figure 1) (11).

Internal reliability was assessed using Cronbach’s alpha on the sample *n* = 243 participants. External reliability was evaluated in a subgroup of participants (*n* = 29) who completed the DIET-SE a second time, four weeks after the initial assessment, and was examined using the intraclass correlation coefficient (ICC) (Figure 1).

Construct validity included convergent validity and related constructs. The Romanian version of the Intuitive Eating Scale-2 (IES-2), validated by Vintilă et al. (10), was used to assess convergent validity. Correlation analyses were conducted between the total DIET-SE score and the total IES-2 score, and also between the total DIET-SE score and the four IES-2 subscales: Unconditional Permission to Eat, Eating for Physical Rather Than Emotional Reasons, Reliance on Hunger and Satiety Cues, and Body–Food Choice Congruence. We expected significant correlations between the questionnaires and between DIET-SE and the relevant IES-2 subscales.

For related constructs validity, the DIET-SE score was tested for associations with socio-demographic variables. We hypothesized that DIET-SE scores would be associated with several participant characteristics, in line with the literature (Figure 1).

### 2.1 Ethics approval

The ethics approval for this research protocol was obtained from the Ethics Committee of Scientific Research of Victor Babes University of Medicine and Pharmacy, Timisoara, Romania, with number 01/05.01.2026. All subjects included in the study provided informed consent before participation.

### 2.2 Data management and data analysis

IBM-SPSS version 21 was used to process the data. Using the original instruction for scoring provided by Stich et al. (5), each item was scored from 0 (not at all confident) to 4 (very confident). The total score was computed by adding the scores of all individual items, resulting in a possible range from 0 to 44. Subscale scores were calculated by summing the corresponding item responses. Higher scores indicated higher dieting self-efficacy.

The IES-2 total and subscale scores were calculated according to the procedure recommended by Vintilă et al. (10), by averaging the relevant item responses. Higher scores indicated higher levels of intuitive eating.

Body mass index (BMI) was calculated as weight in kilograms divided by height in meters squared. Several demographic and health-related variables were recoded prior to the analysis. Self-rated health status was dichotomized into excellent/very good versus all other categories. Age was grouped into three categories: 19–39 years, 40–55 years, and 56–75 years. Discrepancy between perceived and desired body shape was calculated by subtracting the desired body shape score from the perceived body shape score. In all participants, the resulting difference was positive and ranged from 0 to 6. This variable was further categorized into three groups: no discrepancy, 1–2 levels, and 3 or more levels. Average fruit and vegetable intake was also categorized into three groups: up to 2 servings, 3–4 servings, and 5 or more servings. Educational levels were classified as ISCED 4 or less and ISCED 5 or above. Household income levels were dichotomized as below the national household threshold reported by INSS^1^ at 8.832 RON (€1,800) or above this threshold.

Normality of distribution was assessed using the Kolmogorov–Smirnov test. Continuous variables with a normal distribution are presented as means and standard deviations, while categorical variables are presented as percentages. Exploratory factor analysis (EFA) was performed to examine the underlying factor structure of the questionnaire. Prior to factor extraction, the suitability of the data for factor analysis was assessed using the Kaiser–Meyer–Olkin (KMO) measure of sampling adequacy and Bartlett’s test of sphericity. Factors were extracted using principal axis factoring, and varimax rotation was applied to improve interpretability. The number of factors retained was determined based on eigenvalues, scree plot inspection, and factor interpretability.

Confirmatory factor analysis (CFA) was conducted in Stata version 15 (StataCorp LLC, College Station, TX, United States) using the sem procedure. It was performed on an independent subsample to test the factorial structure identified in the EFA. Model parameters were estimated using maximum likelihood, with the five-point Likert-type items treated as approximately continuous variables. Model fit indices were obtained using estat gof command and included several commonly reported fit indices: the chi-square statistic, the comparative fit index (CFI), the Tucker–Lewis index (TLI), the root mean square error of approximation (RMSEA) with its 90% confidence interval, and the standardized root mean square residual (SRMR). Values of CFI and TLI above 0.90 and SRMR and RMSEA values below 0.08 were considered indicative of acceptable model fit.

Internal consistency was assessed using Cronbach’s alpha, and test–retest reliability was evaluated using the intraclass correlation coefficient (ICC). We expected acceptable reliability coefficients, defined as Cronbach’s alpha > 0.70 and ICC > 0.75 (11).

Pearson’s correlation coefficient was used to assess associations between the total and subscale scores of the DIET-SE and IES-2 instruments. Kendall’s tau was used to assess the associations between the DIET-SE total and subscale scores and ordinal variables. Ninety-five percent confidence intervals for Pearson correlation coefficients were calculated using Fisher’s z transformation. Differences in continuous variables were assessed using the independent-samples t-test when comparing two groups and one-way analysis of variance (ANOVA) when comparing three groups.

## 3 Results

A total of 243 participants with BMI ≥ 25 were included in the study. The mean age was 46.1 years (SD = 12.0), and the sample was predominantly female (76.1%). The mean BMI was 29.5 kg/m^2^ (SD = 4.1), with 36.2% of participants classified as having obesity according to WHO criteria. Most participants did not rate their health as excellent or very good (72.8%). Regarding socioeconomic characteristics, 190 participants (78.2%) had completed high school or a lower level of education. Monthly income was below national household average income for 2025 for 143 participants (58.8%) and over this threshold for 79 participants (32.5%), while 21 participants (8.6%) preferred not to disclose their income.

Regarding body image, all participants reported a desired body shape that was either similar to or smaller than their perceived body shape. Of all, 62.1% reported a discrepancy of 1–2 levels between perceived and desired body shape, while 28.4% reported a discrepancy of 3 or more levels. Only 9.5% reported no discrepancy.

Estimated fruit and vegetable intake in the previous 3 days was most commonly 3–4 servings (44.0%) or up to 2 servings (42.8%), whereas only 13.2% reported consuming 5 or more servings. The mean perceived stress level was 5.6 (SD = 2.7) (Table 1).

**Table 1:** Demographic characteristics of the sample (*n* = 243).

Demographics		Count (*N* %)
Gender	Male	58 (23.9%)
	Female	185 (76.1%)
Age categories	19–39 years	78 (32.1%)
	40–55 years	95 (39.1%)
	56–75 years	70 (28.8%)
BMI categories (WHO)	Overweight	155 (63.8%)
	Obesity	88 (36.2%)
Self-perception of health status as excellent or very good	No	177 (72.8%)
	Yes	66 (27.2%)
Discrepancy between perceived and desired body shape	No discrepancy	23 (9.5%)
	1–2 levels	151 (62.1%)
	3 + levels	69 (28.4%)
Estimated average fruit and vegetable intake in last 3 days	Up to 2 servings	104 (42.8%)
	3–4 servings	107 (44.0%)
	5 + servings	32 (13.2%)
Education	ISCED 4 or below	190 (78.2%)
	ISCED 5 or above	53 (21.8%)
Income	Below national household average income for 2025	143 (58.8%)
	National household average income or above for 2025	79 (32.5%)
	Do not want to answer	21 (8.6%)
Age (years)	Mean ± SD	46.1 ± 12.0
BMI (kg/m^2^)	Mean ± SD	29.5 ± 4.1
Perceived stress level	Mean ± SD	5.6 ± 2.7

Harman’s single-factor test showed that the single unrotated factor accounted for 25.08% of the total variance. As this value was substantially below 50%, the analysis did not indicate that a single common method factor was a dominant source of variance in the data.

### 3.1 Structural validity

Factorial validity was assessed using exploratory factor analysis (EFA) and confirmatory factor analysis (CFA) in two independent subsamples (Figure 1). EFA was performed in a random subsample of 123 participants using principal axis factoring with Varimax rotation. The data were adequate for factor analysis, as shown by a Kaiser–Meyer–Olkin value of 0.828 and a significant Bartlett’s test of sphericity (χ^2^ = 562.942, df = 55, *p* < 0.001).

**Figure 2:**
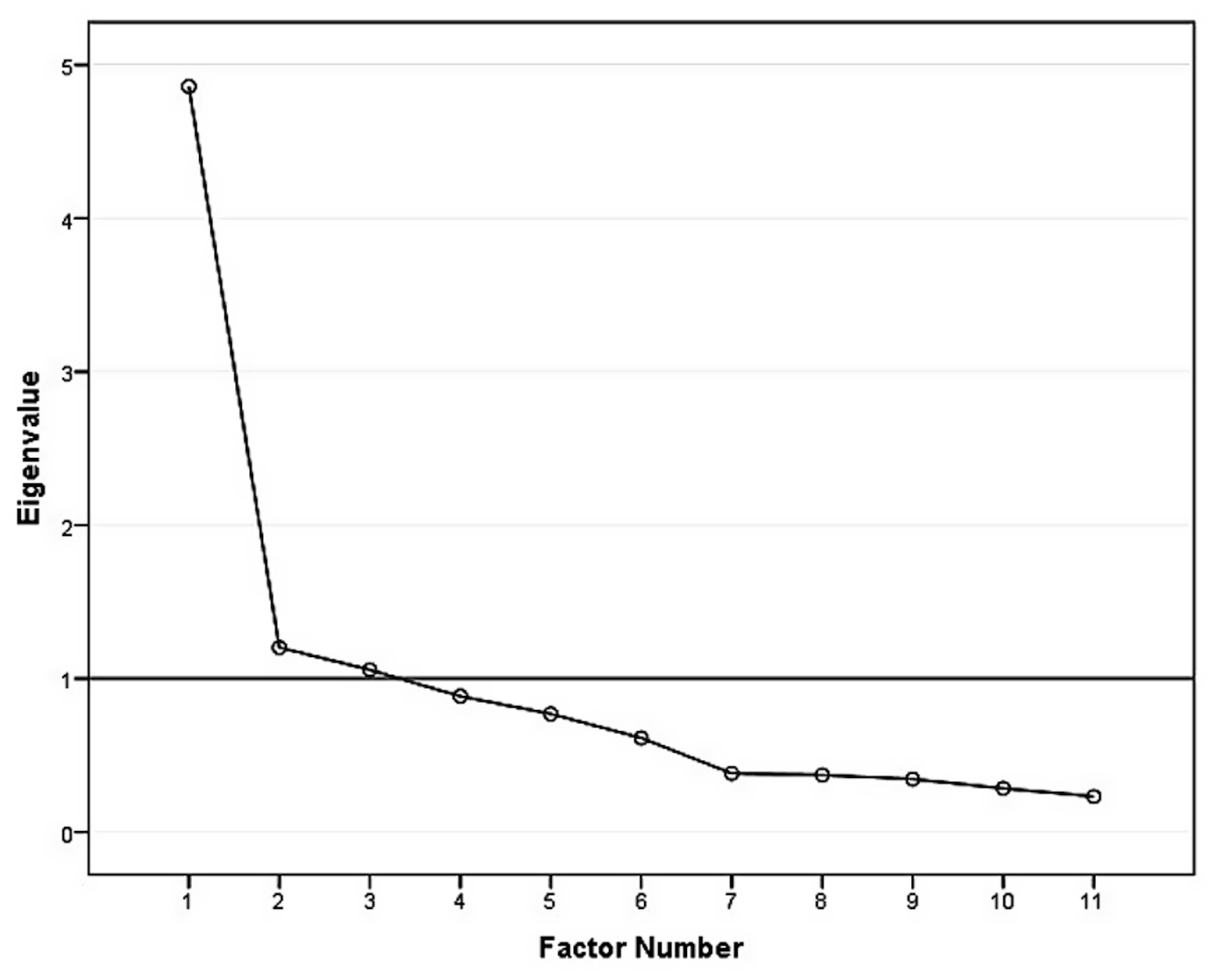
Scree plot of EFA.

A three-factor structure was identified with eigenvalues >1 (Figure 2), consistent with the original scale, explaining 52.9% of the total variance. Factor 1 explained 18.8% of the variance, Factor 2 explained 17.8%, and Factor 3 explained 17.7%.

Table 2 presents the factor loadings after Varimax rotation with Kaiser normalization. The EFA showed a three-factor structure for the Romanian version of the DIET-SE. The first factor, Social and Internal Factors, included 4 items, with loadings between 0.557 and 0.716. The second factor, Negative Emotional Events, included 3 items, with loadings between 0.415 and 0.925. The third factor, High-Caloric Food, included 4 items, with loadings between 0.425 and 0.805. This three-factor structure was consistent with the original instrument. The factor solution was clear and interpretable, and each item showed the highest loading on its corresponding factor.

**Table 2:** Factor loadings after Varimax rotation with Kaiser normalization.

DIET-SE	Structure		
	Social and internal factors	Negative emotional events	High-caloric food
2. You often overeat at supper because you are tired and hungry when you get home. How confident are you that you would not overeat at supper?	**0.716**	0.116	0.278
1. You are having dinner with your family and your favorite meal has been prepared. You finish the first helping and someone says, “Why do not you have some more?” How confident are you that you would turn down a second helping?	**0.677**	0.104	0.177
5. You are invited to someone’s house for dinner and your host is an excellent cook. You often overeat because the food tastes so good. How confident are you that you would not overeat as a dinner guest?	**0.665**	0.366	0.185
9. You feel like celebrating. You are going out with friends to a good restaurant. How confident are you that you would celebrate without overeating?	**0.557**	0.404	0.207
8. You are having a hard day at work and you are anxious and upset. You feel like getting a candy bar. How confident are you that you would find a more constructive way to calm down and cope with your feelings?	0.136	**0.925**	0.208
11. You just had an argument with your boyfriend or girlfriend. You are upset, angry, and you feel like eating something. How confident are you that you would talk the situation over with someone or go for a walk instead of eating?	0.199	**0.570**	0.268
4. You just had an upsetting argument with a family member. You are standing in front of the refrigerator and you feel like eating everything in sight. How confident are you that you would find some other way to make yourself feel better?	0.275	**0.415**	0.184
3. There is a party at work for a coworker and someone offers you a piece of cake. How confident are you that you would turn it down?	0.210	0.251	**0.805**
7. You are at a friend’s house and your friend offers you a delicious looking pastry. How confident are you that you would refuse this offer?	0.241	0.145	**0.771**
10. You are out with a friend at lunch time and your friend suggests that you stop and get some ice cream. How confident are you that you would resist the temptation?	0.275	0.318	**0.441**
6. You finished your meal and you still feel hungry. There are cakes and fruits available. How confident are you that you would choose the fruits?	0.179	0.316	**0.425**

Loadings in bold are values above 0.40.

**Figure 3:**
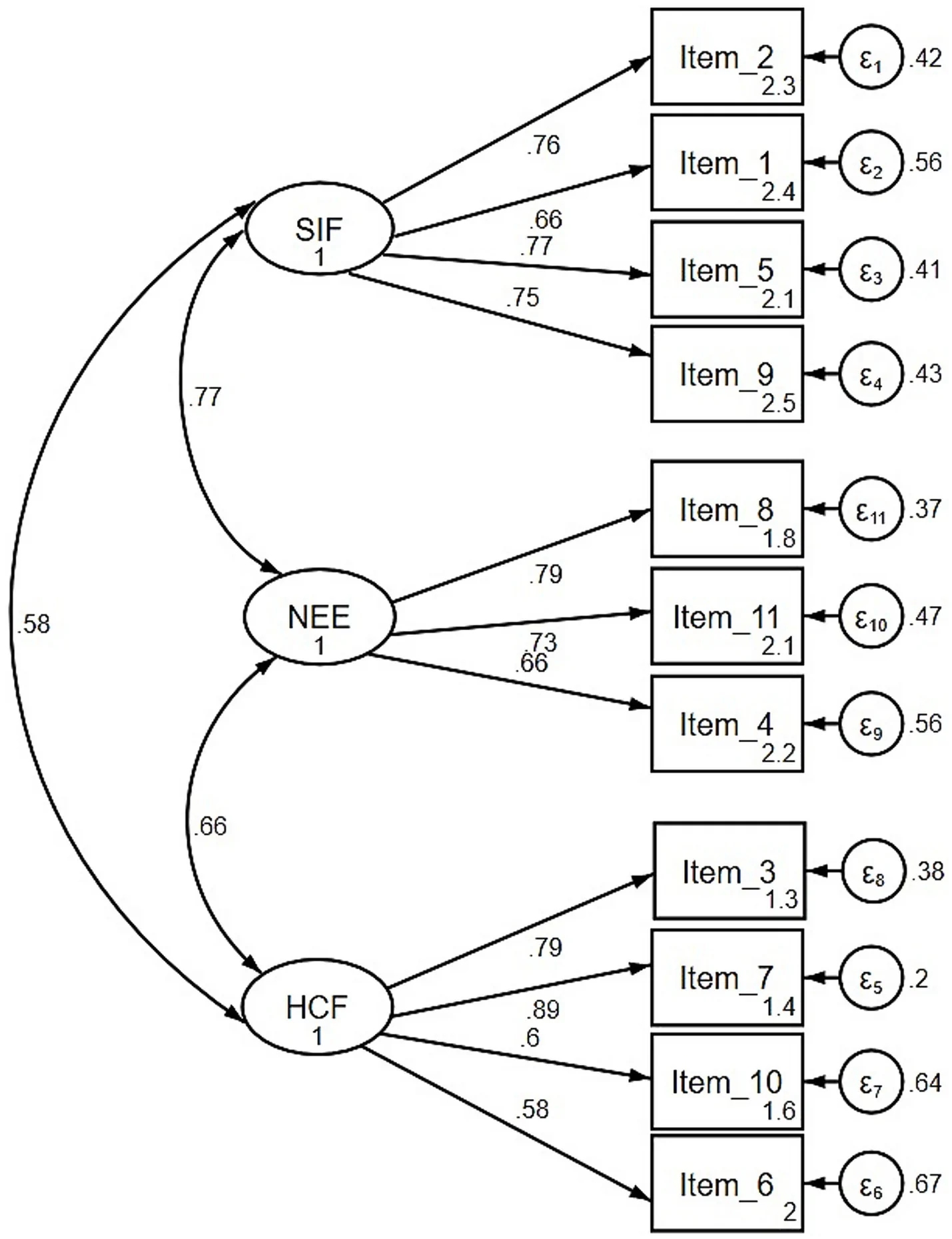
The confirmatory factor analysis of the Romanian version of DIET-SE with a 3-factor structure. Numbers on the double-headed arrows represent the covariance between components, numbers on the single-headed arrows represent the standardized factor loadings.

A confirmatory factor analysis was conducted to test the three-factor model identified in the EFA and consistent with the original instrument. Figure 3 presents the structural equation modeling (SEM) for the three factors that include 11 questions. The CFA model showed a significant chi-square statistic, χ^2^(32) = 62.84, *p* = 0.0009, indicating that the hypothesis of exact model fit was rejected. However, because the chi-square test is a stringent test of exact fit and may be influenced by minor model–data discrepancies, distributional assumptions, and sample characteristics, the model was evaluated using multiple complementary fit indices. The and CFI = 0.935, TLI = 0.908, and SRMR = 0.058 supported an acceptable fit. RMSEA was slightly above the recommended threshold (RMSEA = 0.090, 90% CI: 0.056–0.122; *p*-close = 0.028), indicating some residual model misfit. All items loaded on their expected latent factors, providing additional support for the proposed three-factor structure. Covariances between latent factors are positive and significant. Taken together, these findings provided qualified support for the proposed three-factor structure, although the model fit was not unequivocal.

### 3.2 Reliability

Internal reliability (Figure 1) was good, with a Cronbach’s alpha of 0.872 for the 11-item Romanian version of the DIET-SE. The standardized Cronbach’s alpha was very similar (0.873), indicating good internal consistency of the scale. Item-total statistics further supported the internal consistency of the Romanian DIET-SE. Cronbach’s alpha if item deleted ranged from 0.855 to 0.869, showing that removal of any item would not improve the internal consistency of the instrument. The three subscales also showed acceptable to good internal consistency: Cronbach’s alpha was 0.834 for Social and Internal Factors (4 items), 0.777 for Negative Emotional Events (3 items) and 0.763 for High-Caloric Food (4 items).

Test–retest reliability (Figure 1) was excellent, with a single-measures ICC of 0.942 (95% CI: 0.880–0.972, *p* < 0.001).

### 3.3 Construct validity

Convergent validity (Figure 1) was assessed using Pearson correlations between the DIET-SE scores and the Intuitive Eating Scale-2 (IES-2) total and subscale scores. As shown in Table 2, the DIET-SE dimensions were positively correlated with the IES-2 total score, with coefficients ranging from 0.219 to 0.413 (all *p* < 0.01). Significant positive correlations were also found with the IES-2 subscales Reliance on Hunger and Satiety Cues and Body–Food Choice Congruence. In contrast, no significant associations were observed with Unconditional Permission to Eat or Eating for Physical Rather than Emotional Reasons. The pattern of small-to-moderate correlations supports the convergent validity of the Romanian version of the DIET-SE (see Table 3).

**Table 3:** Correlations between the Romanian DIET-SE and the intuitive eating scale-2 total and subscale scores (*n* = 243).

IES-2	DIET-SE total	High-caloric food	Social/internal	Negative emotional events
Total	**0.401** [0.290, 0.502]	**0.219** [0.096, 0.336]	**0.375** [0.261, 0.478]	**0.413** [0.303, 0.512]
Unconditional permission to eat	0.049 [−0.077, 0.174]	0.111 [−0.015, 0.234]	−0.010 [−0.136, 0.116]	0.016 [−0.110, 0.142]
Eating for physical rather than emotional reasons	0.022 [−0.104, 0.147]	−0.106 [−0.229, 0.020]	0.070 [−0.056, 0.194]	0.105 [−0.021, 0.228]
Reliance on hunger and satiety cues	**0.389** [0.277, 0.491]	**0.276** [0.156, 0.388]	**0.329** [0.212, 0.437]	**0.370** [0.256, 0.474]
Body–food choice congruence	**0.327** [0.210, 0.435]	**0.183** [0.059, 0.302]	**0.335** [0.218, 0.442]	**0.297** [0.178, 0.408]

Pearson correlation r in bold are statistically significant *p* < 0.05.

### 3.4 Associations with related constructs

Table 4 shows the mean DIET-SE total and subscale scores according to participant characteristics. No significant differences were found by gender for the total score or any of the subscales. Likewise, no significant differences were observed across age categories. In contrast, significant differences were found according to BMI category. Participants with overweight had higher DIET-SE total scores than those with obesity (24.2 ± 8.9 vs. 21.4 ± 8.4, *p* = 0.017). They also had higher scores on the Social and Internal Factors subscale (9.9 ± 3.7 vs. 8.5 ± 3.9, *p* = 0.004) and the Negative Emotional Events subscale (7.0 ± 3.1 vs. 5.8 ± 3.1, *p* = 0.004), while no significant difference was found for the High-Caloric Food subscale (*p* = 0.701).

**Table 4:** Associations of scores of DIET-SE and subscales with related constructs.

Related constructs		DIET-SE	DIET-SE subscale: high-caloric food	DIET-SE subscale: social and internal factors	DIET-SE subscale: negative emotional events
Gender	Male	23.3 (8.3)	7.5 (3.8)	8.8 (3.9)	7.0 (2.9)
	Female	23.1 (9.0)	7.1 (3.7)	9.6 (3.7)	6.5 (3.2)
	*p*-value *	0.892	0.467	0.183	0.259
Age categories	19–39 years	23.8 (9.0)	7.0 (3.8)	9.7 (3.8)	7.1 (3.1)
	40–55 years	22.3 (8.8)	7.3 (3.7)	8.8 (3.8)	6.2 (3.2)
	56–75 years	23.7 (8.6)	7.2 (3.8)	10.0 (3.6)	6.5 (3.0)
	*p*-value **	0.471	0.838	0.096	0.142
BMI categories	Overweight	24.2 (8.9)	7.2 (3.8)	9.9 (3.7)	7.0 (3.1)
	Obesity	21.4 (8.4)	7.0 (3.7)	8.5 (3.9)	5.8 (3.1)
	*p*-value *	**0.017**	0.701	**0.004**	**0.004**
Self-perception of health status as excellent or very good	No	22.4 (8.9)	6.9 (3.8)	9.2 (3.7)	6.3 (3.2)
	Yes	25.2 (8.4)	7.8 (3.7)	10.1 (3.9)	7.3 (2.8)
	*p*-value *	**0.027**	0.093	0.094	**0.030**
Perceived stress level (10 levels: higher levels, higher stress level)	tau	**−0.169** ^**^	−0.116	**−0.137** ^*^	**−0.192** ^**^
	*p*-value ***	0.008	0.070	0.033	0.003
Body mass index	kg/m^2^	**−0.160** ^*^	−0.015	**−0.198** ^**^	**−0.186** ^**^
	*p*-value ***	0.012	0.814	0.002	0.004
Perceived body shape (9 level: Higher level bigger body size)	tau	**−0.286** ^**^	**−0.172** ^**^	**−0.277** ^**^	**−0.284** ^**^
	*p*-value ***	<0.001	0.007	<0.001	<0.001
Desired body shape (9 level: Higher level bigger body size)	tau	**−0.162** ^*^	**−0.137** ^*^	**−0.160** ^*^	**−0.138** ^*^
	*p*-value ***	0.011	0.033	0.012	0.031
Discrepancy between perceived and desired body shape (7 levels: from no discrepancy to 6 levels of discrepancy)	tau	**−0.214** ^**^	−0.092	**−0.194** ^**^	**−0.240** ^**^
	*p*-value ***	0.001	0.151	0.002	<0.001
Estimated average fruit and vegetable intake in last 3 days (6 level: higher level, higher intake)	tau	**0.160** ^*^	**0.139** ^*^	0.096	**0.141** ^*^
	*p*-value ***	0.012	0.030	0.138	0.028

*t-test, ** ANOVA, *** Kendall tau. Values in bold are statistically significant *p* < 0.05.

Significant differences were also observed according to self-perceived health status. Participants who rated their health as excellent or very good had higher total DIET-SE scores than those who did not (25.2 ± 8.4 vs. 22.4 ± 8.9, *p* = 0.027). They also had higher scores on the Negative Emotional Events subscale (7.3 ± 2.8 vs. 6.3 ± 3.2, *p* = 0.030). Differences for the High-Caloric Food and Social and Internal Factors subscales did not reach statistical significance (*p* = 0.093 and *p* = 0.094, respectively).

Table 4 also presents the correlations between DIET-SE scores and several related variables. Perceived stress level was negatively correlated with the total DIET-SE score (r = −0.169, *p* = 0.008), as well as with the Social and Internal Factors (r = −0.137, *p* = 0.033) and Negative Emotional Events (r = −0.192, *p* = 0.003) subscales. No significant correlation was found with the High-Caloric Food subscale (*p* = 0.070).

BMI was also negatively correlated with the total DIET-SE score (r = −0.160, *p* = 0.012), the Social and Internal Factors subscale (r = −0.198, *p* = 0.002), and the Negative Emotional Events subscale (r = −0.186, *p* = 0.004), but not with the High-Caloric Food subscale (*p* = 0.814).

Perceived body shape showed significant negative correlations with the total score and all three subscales, with coefficients ranging from −0.172 to −0.286 (all *p* < 0.01). Desired body shape was also negatively correlated with the total DIET-SE score and all subscales, although these associations were weaker, with coefficients ranging from −0.137 to −0.162 (all *p* < 0.05).

The discrepancy between perceived and desired body shape was negatively correlated with the total DIET-SE score (r = −0.214, *p* = 0.001), the Social and Internal Factors subscale (r = −0.194, *p* = 0.002), and the Negative Emotional Events subscale (r = −0.240, *p* < 0.001), but not with the High-Caloric Food subscale (*p* = 0.151).

Finally, estimated average fruit and vegetable intake in the last 3 days was positively correlated with the total DIET-SE score (r = 0.160, *p* = 0.012), the High-Caloric Food subscale (r = 0.139, *p* = 0.030), and the Negative Emotional Events subscale (r = 0.141, *p* = 0.028), while the correlation with the Social and Internal Factors subscale was not statistically significant (*p* = 0.138).

## 4 Discussion

This study examined the psychometric properties of the Romanian version of the DIET-SE in adults with overweight and obesity. The findings suggest that the instrument has adequate psychometric performance in this population and may be useful for assessing perceived confidence in handling eating-related situations. More broadly, the results support the view that self-efficacy is an important self-regulatory resource in weight-related behaviour change. In adults with overweight or obesity, self-regulation processes, including self-efficacy, have consistently been associated with dietary change and longer-term weight management (1, 12).

The factor structure of the Romanian version was broadly consistent with the main conceptual organization of the original instrument (5), similar to validations in Polish (6), Mexican Spanish (7), and Turkish (8). This is important because it suggests that the Romanian version reflects the same core dimensions of dieting self-efficacy as the original instrument. In turn, this supports the cross-cultural relevance of the scale and increases confidence that the Romanian adaptation preserved the intended meaning of the construct.

The high EFA loading of Item 8 indicates a particularly strong association with the Negative Emotional Events factor, but does not necessarily imply redundancy, as the item captures a distinct work-related emotional eating context. Items 6 and 10 showed lower but acceptable loadings above 0.40 and were retained because they loaded most strongly on their intended factor and contributed to the content coverage of the original scale. An interesting finding was the different order of factor emergence compared with the original study. While the original version identified High-Calorie Food first, followed by Social and Internal Factors and Negative Emotional Events, in our sample, the factors emerged as Social and Internal Factors, Negative Emotional Events, and High-Calorie Food. This difference should not be interpreted as evidence of a different underlying structure, because in exploratory factor analysis, factor order mainly reflects the amount of variance explained in the specific sample (11). However, it may suggest that, in Romanian adults with excess weight, social/internal cues and emotionally challenging situations were relatively more salient in shaping dieting self-efficacy than high-calorie food cues alone. This interpretation is also consistent with previous findings in Romanian adults (13, 14) showing that less adaptive eating-related profiles were associated with excess weight, higher stress, and poorer self-rated health, supporting the idea that, in this population, eating difficulties may be embedded in a broader self-regulatory and psychosocial context rather than being driven only by food exposure itself. Broader evidence shows that emotional and contextual eating patterns are especially relevant in overweight and obesity (15, 16).

The Cronbach’s alpha analysis indicated that the Romanian DIET-SE was a reliable instrument in this sample. The internal consistency of the DIET-SE subscales ranged from 0.763 to 0.834, suggesting acceptable to good homogeneity of the items within each factor for measuring dieting self-efficacy across specific eating-related situations. The Cronbach’s alpha for the total scale was 0.872, in line with results reported for the original instrument (Cronbach’s alpha = 0.82) (5), as well as for the Polish (Cronbach’s alpha = 0.79) (6), Mexican Spanish (Cronbach’s alpha = 0.77) (7), and Turkish versions (Cronbach’s alpha = 0.787) (8). Test–retest reliability over a four-week interval showed adequate temporal stability, with an overall ICC of 0.942, which is considered high, indicating good stability.

The convergent validity findings suggest that DIET-SE is more strongly aligned with the self-regulatory and body-attuned dimensions of adaptive eating than with all components of intuitive eating equally. In our study, the DIET-SE total score and all three subscales were positively associated with the IES-2 total score, as well as with Reliance on Hunger and Satiety Cues and Body-Food Choice Congruence, supporting the assumption that greater dieting self-efficacy is related to a more internally regulated and health-oriented eating style. These associations provide complementary evidence of convergent validity. The interpretation is consistent with broader evidence showing that intuitive eating is generally associated with more favorable psychological and behavioural outcomes, and that its strongest links with diet quality and adaptive eating tend to involve greater sensitivity to internal cues and food choices that are congruent with bodily needs (17–19).

By contrast, the lack of significant associations with Unconditional Permission to Eat and Eating for Physical Rather than Emotional Reasons may reflect true conceptual differences rather than weak convergent validity. DIET-SE assesses confidence in managing challenging eating situations, such as exposure to palatable foods, social pressure, or negative emotional contexts, whereas these two IES-2 subscales may capture somewhat different processes, including reduced dietary rigidity and the motives underlying eating episodes. Previous research suggests that the IES-2 subscales do not behave uniformly. The Unconditional Permission to Eat has sometimes shown more complex and even less favourable associations with diet quality than the other intuitive eating dimensions when examined separately (18, 20, 21).

This pattern supports partial but meaningful convergent validity of the Romanian DIET-SE scale. Rather than overlapping with intuitive eating globally, DIET-SE appears to converge most clearly with those aspects of intuitive eating that reflect trust in internal appetite signals and food choices made in accordance with bodily functioning, which is theoretically coherent with a construct centred on self-efficacy in dietary self-regulation (17, 18, 22).

No significant associations were found between age or gender and the DIET-SE total score or its subscales in this sample with overweight and obesity. This finding may be explained by the fact that dieting self-efficacy is conceptualized as a situation-specific belief in one’s ability to manage specific eating challenges, rather than as a construct inherently determined by demographic characteristics. The DIET-SE was specifically designed to capture confidence in coping with high-calorie food temptations, social/internal triggers, and negative emotional events (5). This is consistent with social cognitive theory, which describes self-efficacy as behaviours-specific and primarily shaped by experience and contextual demands (3). Previous literature indicates that diet-related self-efficacy is a context-sensitive construct and that demographic differences may vary across populations and study settings. Therefore, our findings may reflect the fact that perceived ability to manage challenging eating situations depends more on individual behavioural experience and self-regulatory resources than on age or gender alone (4, 23, 24).

The associations with BMI and body-image-related variables were also conceptually coherent. Participants with obesity had lower DIET-SE scores than those with overweight, and lower dieting self-efficacy was related to a larger perceived body shape, a larger desired body shape, and to a greater discrepancy between them. This pattern is compatible with review evidence showing that self-efficacy is a key self-regulation mechanism in obesity treatment (1) and with meta-analytic findings that dietary self-efficacy is modifiable through behaviour-change interventions (4). This finding may indicate that body dissatisfaction and reduced confidence in managing eating-related challenges coexist, although the cross-sectional design does not allow causal interpretation. Previous intervention studies have separately linked improvements in body image with better eating self-regulation and changes in dietary self-efficacy with weight-related outcomes (25, 26).

The associations with perceived stress and self-rated health were also conceptually coherent. Participants reporting excellent or very good health had higher DIET-SE scores, whereas higher perceived stress was associated with lower dieting self-efficacy. This pattern suggests that in population with excess weight, lower dieting self-efficacy may coexist both with greater psychological strain and with a less favourable subjective perception of health. This interpretation is supported by review evidence identifying self-efficacy as a key self-regulation mechanism in weight management, by meta-analytic findings showing that stress-management components can improve dietary self-efficacy, and by evidence that behavioural weight-management interventions can improve health-related quality of life (4, 27, 28).

The positive association between DIET-SE and fruit and vegetable intake further supports the behavioural relevance of the construct. Although modest in magnitude, this finding suggests that higher dieting self-efficacy may be reflected in healthier food choices in daily life. This interpretation is consistent with review evidence identifying self-efficacy as a robust predictor of adult fruit and vegetable intake (29) and with intervention research showing that dietary behaviours can be improved through approaches targeting self-efficacy and related behaviour-change mechanisms (30, 31). This result is also clinically meaningful in Romanian adults with obesity, as previous findings from our group suggest that implementing dietary recommendations in daily life remains challenging even under medical supervision (32). In this context, higher DIET-SE may reflect a greater perceived capacity to translate dietary goals into healthier food choices, including fruit and vegetable intake.

Several limitations should still be acknowledged. The study was based on self-reported measures, which may introduce reporting bias, and the sample included only adults with overweight or obesity, so the findings should not be generalized beyond similar populations without caution. Although the CFA subsample met the commonly used criterion of at least 10 participants per observed item, replication in a larger independent sample would provide additional confirmation of the precision and stability of the three-factor solution. The five-point Likert-type items were treated as approximately continuous in the CFA, which may have influenced some model-fit indices, particularly RMSEA. In addition, some fit indices from the CFA were acceptable rather than strong, which suggests that the factorial structure is supported, but not without reservation. Future studies could examine the scale in broader Romanian samples and test its usefulness in longitudinal or intervention contexts. The assessment of convergent validity relied on the Romanian IES-2, which has reported psychometric limitations; therefore, the observed correlations should not be regarded as definitive evidence of convergent validity. Measurement invariance across gender was not assessed because of the small and unbalanced male subgroup.

In conclusion, the Romanian DIET-SE demonstrated satisfactory validity and reliability in adults with excess weight. The findings support its usefulness as a tool for assessing confidence in managing challenging eating situations, while also suggesting that the relative salience of dieting difficulties may vary across populations. Its associations with clinically and behaviourally relevant variables further support the construct validity of the scale. The Romanian DIET-SE may therefore be useful in both research and clinical contexts focused on eating behaviours and weight management.

## Data Availability

The raw data supporting the conclusions of this article will be made available by the authors, without undue reservation.

## Footnotes

1
www.inss.ro
