# Supplementary material for: Psychometric validation of the Romanian Scenario-Based Dieting Self-Efficacy Scale in adults with overweight and obesity
Source: Front Nutr. 2026 Sep 1;13:1911825. doi: 10.3389/fnut.2026.1911825 (PMC13574676; doi:10.3389/fnut.2026.1911825)
Supplement: Supplementary file 1 [file Table_1.docx]

Supplementary Material

# Draia et al. Psychometric validation of the Romanian Scenario-Based Dieting Self-Efficacy Scale in adults with overweight and obesity

# Supplementary Table 1. English and Romanian version of the Scenario-Based Dieting Self-Efficacy Scale

|  | **SE-DIET English** | **SE-DIET Romanian** |
| --- | --- | --- |
|  | *Response options for all items: 0 = not at all confident; 1 = slightly confident; 2 = moderately confident; 3 = quite confident; 4 = very confident.* | *Opțiuni de răspuns pentru toți itemii: 0 = deloc încrezător; 1 = puțin încrezător; 2 = moderat încrezător; 3 = destul de încrezător; 4 = foarte încrezător.* |
| 1. | You are having dinner with your family and your favorite meal has been prepared. You finish the first helping and someone says, “Why don’t you have some more?” How confident are you that you would turn down a second helping | Sunteți la cină cu familia, și aveți pe masă felul d-voastre preferat de mâncare. Ați terminat prima porție, iar cineva vă spune: „Hai, mai ia puțin!”. Cât de încrezător sunteți că ați putea refuza o a doua porție? |
| 2. | You often overeat at supper because you are tired and hungry when you get home. How confident are you that you would not overeat at supper? | Cât de încrezător sunteți că ați reuși să nu mâncați în exces la cină, chiar dacă sunteți obosit(ă) și flămând(ă)? |
| 3. | There is a party at work for a coworker and someone offers you a piece of cake. How confident are you that you would turn it down? | La serviciu are loc o petrecere pentru un coleg, iar cineva vă oferă o felie de tort. Cât de încrezător sunteți că ați refuza tortul? |
| 4. | You just had an upsetting argument with a family member. You are standing in front of the refrigerator and you feel like eating everything in sight. How confident are you that you would find some other way to make yourself feel better? | Tocmai ați avut o ceartă supărătoare cu un membru al familiei. Stați în fața frigiderului și simțiți nevoia să mâncați tot ce vedeți. Cât de încrezător sunteți că ați găsi o altă modalitate de a vă simți mai bine? |
| 5. | You are invited to someone’s house for dinner and your host is an excellent cook. You often overeat because the food tastes so good. How confident are you that you would not overeat as a dinner guest? | Sunteți invitat(ă) la cină la cineva acasă, iar gazda este un bucătar excelent. De obicei mâncați prea mult pentru că mâncarea este foarte gustoasă.  Cât de încrezător sunteți că nu ați mânca în exces ca oaspete la cină? |
| 6. | You finished your meal and you still feel hungry. There are cakes and fruits available. How confident are you that you would choose the fruits? | Ați terminat masa și încă vă este foame. Sunt disponibile prăjituri și fructe. Cât de încrezător sunteți că ați alege fructele? |
| 7. | You are at a friend’s house and your friend offers you a delicious looking pastry. How confident are you that you would refuse this offer? | Sunteți în vizită la un prieten, iar acesta vă oferă o prăjitură care arată delicios.  Cât de încrezător sunteți că ați refuza această ofertă? |
| 8. | You are having a hard day at work and you are anxious and upset. You feel like getting a candy bar. How confident are you that you would find a more constructive way to calm down and cope with your feelings? | Aveți o zi grea la serviciu și vă simțiți anxios/anxioasă și supărat(ă). Vă vine să luați o tabletă de ciocolată.  Cât de încrezător sunteți că ați găsi o modalitate mai constructivă de a vă calma și gestiona emoțiile? |
| 9. | You feel like celebrating. You are going out with friends to a good restaurant. How confident are you that you would celebrate without overeating? | Sunteți într-o dispoziție de sărbătoare. Ieșiți cu prietenii la un restaurant bun.  Cât de încrezător sunteți că ați sărbători fără să mâncați în exces? |
| 10. | You are out with a friend at lunch time and your friend suggests that you stop and get some ice cream. How confident are you that you would resist the temptation? | Sunteți pe stradă cu un prieten, iar acesta vă propune să vă opriți să luați o înghețată. Cât de încrezător sunteți că ați rezista tentației? |
| 11. | You just had an argument with your boy- friend or girlfriend. You are upset, angry, and you feel like eating something. How confident are you that you would talk the situation over with someone or go for a walk instead of eating? | Tocmai ați avut o ceartă cu partenerul(a). Sunteți supărat(ă), furios/furioasă și simțiți nevoia să mâncați ceva.  Cât de încrezător sunteți că ați discuta situația cu cineva sau ați merge la o plimbare în loc să mâncați? |
